# Improved Detection of Mild Cognitive Impairment From Temporal Language Markers: I-CONECT Study

**DOI:** 10.1093/geroni/igaf122.1205

**Published:** 2025-12-31

**Authors:** Jiayu Zhou, Siqi Liang, Yijiang Pang, Hiroko Dodge, Bao Hoang

**Affiliations:** University of Michigan, Ann Arbor, Michigan, United States; University of Michigan, Ann Arbor, Michigan, United States; Michigan State University, East Lansing, Michigan, United States; Massachusetts General Hospital, Harvard Medical School, Boston, Massachusetts, United States; Michigan State University, East Lansing, Michigan, United States

## Abstract

**Background:**

Mild Cognitive Impairment (MCI) is an early stage of Alzheimer’s disease, where timely detection can significantly improve intervention outcomes and quality of life. Language markers from routine conversations offer a promising, accessible method to identify MCI. Current research primarily aggregates multiple conversations, potentially masking valuable dynamic cognitive fluctuations over time. Additionally, individual differences in speech styles complicate cognitive assessments. We address this by proposing a novel “temporal harmonization” method, enhancing MCI detection accuracy through personalized language analysis.

**Method:**

Using 6,771 conversation samples from 74 older adults participating in the Internet-Based Conversational Engagement Clinical Trial (I-CONECT, ClinicalTrials.gov#: NCT02871921), we analyzed linguistic indicators including vocabulary diversity, grammatical complexity, and conversational response patterns collected monthly over 12 months. Our innovative harmonization technique leverages advanced machine learning methods to distinguish cognitive changes from personal speaking styles, thus increasing the accuracy and reliability of detecting early cognitive impairment.

**Result:**

Our analysis demonstrates that assessing language markers as temporal sequences significantly improves MCI detection accuracy compared to traditional, single-conversation methods. With temporal harmonization, the predictive accuracy increased notably (AUC=0.721 vs. 0.648 without harmonization), underscoring the value of considering personalized conversational patterns over time.

**Conclusion:**

Routine conversational language patterns analyzed longitudinally can effectively signal early cognitive impairment. Our temporal harmonization method further improves predictive precision by controlling individual speaking differences. Such conversational screening holds potential as a practical, non-intrusive, and ecologically valid tool for social workers and geriatric professionals supporting cognitive health in older adults.

